# Daratumumab, bortezomib, and dexamethasone in relapsed or refractory multiple myeloma: subgroup analysis of CASTOR based on cytogenetic risk

**DOI:** 10.1186/s13045-020-00948-5

**Published:** 2020-08-20

**Authors:** Katja Weisel, Andrew Spencer, Suzanne Lentzsch, Hervé Avet-Loiseau, Tomer M. Mark, Ivan Spicka, Tamas Masszi, Birgitta Lauri, Mark-David Levin, Alberto Bosi, Vania Hungria, Michele Cavo, Je-Jung Lee, Ajay Nooka, Hang Quach, Markus Munder, Cindy Lee, Wolney Barreto, Paolo Corradini, Chang-Ki Min, Asher A. Chanan-Khan, Noemi Horvath, Marcelo Capra, Meral Beksac, Roberto Ovilla, Jae-Cheol Jo, Ho-Jin Shin, Pieter Sonneveld, Tineke Casneuf, Nikki DeAngelis, Himal Amin, Jon Ukropec, Rachel Kobos, Maria-Victoria Mateos

**Affiliations:** 1 Department of Oncology, Hematology and Bone Marrow Transplantation with Section of Pneumology, University Medical Center Hamburg-Eppendorf, Hamburg, Germany; 2 Malignant Haematology and Stem Cell Transplantation Service, Alfred Health-Monash University, Melbourne, Australia; 3 Division of Hematology/Oncology, Columbia University, New York, NY USA; 4 Unite de Genomique du Myelome, CHU Rangueil, Toulouse, France; 5 Department of Medicine, University of Colorado, Aurora, CO USA; 6 Clinical Department of Haematology, 1st Medical Department, Charles University in Prague, Prague, Czech Republic; 7 László Hospital, 3rd Department of Internal Medicine, Semmelweis University, Budapest, Hungary; 8 Department of Hematology, Sunderbyn Hospital, Luleå, Sweden; 9 Department of Internal Medicine, Albert Schweitzer Hospital, Dordrecht, The Netherlands; 10 Department of Hematology, Careggi Hospital and University of Florence, Firenze, Italy; 11 Irmandade Da Santa Casa De Misericordia De São Paulo, São Paulo, Brazil; 12 “Seràgnoli” Institute of Hematology, Department of Experimental, Diagnostic and Specialty Medicine, University of Bologna, Bologna, Italy; 13 Department of Hematology-Oncology, Chonnam National University Hwasun Hospital, Hwasun, Jeollanamdo South Korea; 14 Winship Cancer Institute, Emory University, Atlanta, GA USA; 15 University of Melbourne, St Vincent’s Hospital, Melbourne, Australia; 16 University Medical Center of the Johannes Gutenberg University, Third Department of Medicine, Mainz, Germany; 17 Royal Adelaide Hospital, North Terrace, Adelaide, Australia; 18 University of São Paulo, Ribeirão Preto, Brazil; 19 Fondazione IRCCS Istituto Nazionale dei Tumori, University of Milan, Milan, Italy; 20 Seoul St. Mary’s Hospital, Seoul, South Korea; 21 Mayo Clinic Florida, Jacksonville, FL USA; 22 Instituto do Cancer-Hospital Mae de Deus, Porto Alegre, Brazil; 23 Ankara University, Ankara, Turkey; 24 Hospital Angeles Lomas, Naucalpan de Juárez y alrededores, Mexico; 25 Ulsan University Hospital, Ulsan, South Korea; 26 Department of Internal Medicine, Pusan National University Hospital, Busan, South Korea; 27 Erasmus MC, Rotterdam, The Netherlands; 28 Janssen Research & Development, LLC, Beerse, Belgium; 29 Janssen Research & Development, LLC, Spring House, PA USA; 30 Janssen Research & Development, LLC, Raritan, NJ USA; 31 Janssen Global Scientific Affairs, Horsham, PA USA; 32 University Hospital of Salamanca/IBSAL/Cancer Research Center—IBMCC (USAL-CSIC), Salamanca, Spain

**Keywords:** Clinical trials, Multiple myeloma, Myeloma therapy

## Abstract

**Background:**

Multiple myeloma (MM) patients with high cytogenetic risk have poor outcomes. In CASTOR, daratumumab plus bortezomib/dexamethasone (D-Vd) prolonged progression-free survival (PFS) versus bortezomib/dexamethasone (Vd) alone and exhibited tolerability in patients with relapsed or refractory MM (RRMM).

**Methods:**

This subgroup analysis evaluated D-Vd versus Vd in CASTOR based on cytogenetic risk, determined using fluorescence in situ hybridization and/or karyotype testing performed locally. High-risk patients had t(4;14), t(14;16), and/or del17p abnormalities. Minimal residual disease (MRD; 10^−5^ sensitivity threshold) was assessed via the clonoSEQ® assay V2.0. Of the 498 patients randomized, 40 (16%) in the D-Vd group and 35 (14%) in the Vd group were categorized as high risk.

**Results:**

After a median follow-up of 40.0 months, D-Vd prolonged median PFS versus Vd in patients with standard (16.6 vs 6.6 months; HR, 0.26; 95% CI, 0.19-0.37; *P* < 0.0001) and high (12.6 vs 6.2 months; HR, 0.41; 95% CI, 0.21–0.83; *P* = 0.0106) cytogenetic risk. D-Vd achieved deep responses, including higher rates of MRD negativity and sustained MRD negativity versus Vd, regardless of cytogenetic risk. The safety profile was consistent with the overall population of CASTOR.

**Conclusion:**

These updated data reinforce the effectiveness and tolerability of daratumumab-based regimens for RRMM, regardless of cytogenetic risk status.

**Trial registration:**

ClinicalTrials.gov, NCT02136134. Registered 12 May 2014

## Background

Daratumumab is a human IgGκ monoclonal antibody targeting CD38 with a direct on-tumor [1–4] and immunomodulatory mechanism of action [5–7]. Intravenous daratumumab 16 mg/kg is approved as monotherapy in patients with heavily pre-treated relapsed or refractory multiple myeloma (RRMM) and in combination with bortezomib/dexamethasone (Vd) or lenalidomide/dexamethasone (Rd) in patients with multiple myeloma (MM) who received at least 1 prior line of therapy and in combination with pomalidomide/dexamethasone in patients with at least 2 prior therapies, including lenalidomide and a proteasome inhibitor [8]. Daratumumab is also approved in combination with bortezomib/melphalan/prednisone and in combination with Rd in patients with transplant-ineligible newly diagnosed MM, and in combination with bortezomib/thalidomide/dexamethasone in patients with transplant-eligible newly diagnosed MM [8].

In the primary analysis of the phase 3 CASTOR study of daratumumab plus Vd (D-Vd) versus Vd alone in patients with RRMM, at a median follow-up of 7.4 months, D-Vd significantly prolonged progression-free survival (PFS) and increased rates of minimal residual disease (MRD) negativity and demonstrated a tolerable safety profile [9, 10]. With more than 3 years of follow-up (median 40.0 months) and compared with patients receiving Vd only, patients receiving D-Vd demonstrated a 69% reduction in the risk of disease progression or death (median PFS, 16.7 months vs 7.1 months; hazard ratio [HR], 0.31; 95% confidence interval [CI], 0.25–0.40; *P* < 0.0001); showed significantly better overall response rates (85% vs 63%; *P* < 0.0001); and achieved better rates of complete response (CR) or better (30% vs 10%; *P* < 0.0001), very good partial response (VGPR) or better (63% vs 29%; *P* < 0.0001), and MRD negativity at the 10^−5^ sensitivity threshold (14% vs 2%; *P* < 0.000001) [11]. Patients who received 1 prior line of therapy demonstrated the greatest benefit with D-Vd, including a 78% reduction in the risk of disease progression or death versus Vd (median PFS, 27.0 months vs 7.9 months; HR, 0.22; 95% CI, 0.15–0.32; *P* < 0.0001) and a response of CR or better (43% vs 15%; *P* < 0.0001) and MRD negativity (10^−5^; 20% vs 3%; *P* = 0.000025). In CASTOR, no new safety concerns were observed with longer follow-up [11].

Patients with MM and specific cytogenetic markers are at higher risk for poor outcomes [12, 13]. The International Myeloma Working Group recommends defining high cytogenetic risk as testing positive for at least 1 of the following abnormalities: t(4;14), t(14;16), or del17p, determined by fluorescence in situ hybridization (FISH) [14]. This subgroup analysis of CASTOR presents updated efficacy and safety findings for D-Vd versus Vd treatment based on cytogenetic risk status after a median follow-up of 40.0 months.

## Methods

### Patients

Complete study methodology and primary results from CASTOR have been previously described [9, 15]. Briefly, eligible patients received at least 1 prior line of MM therapy, with at least a partial response to at least 1 prior MM therapy, and had documented progressive disease during or after their last regimen, as defined by the International Myeloma Working Group criteria [16, 17]. Key exclusion criteria included the following: creatinine clearance ≤ 20 mL/min/1.73 m^2^ body surface area, disease refractory or intolerant to bortezomib, disease refractory to a different proteasome inhibitor, or presence of grade ≥ 2 peripheral neuropathy or neuropathic pain.

### Study design and treatment

CASTOR is a multicenter, randomized, open-label, active-controlled, phase 3 trial enrolling patients with RRMM. Randomization was stratified by the International Staging System (stage I, II, or III) at screening, the number of prior lines of therapy (1 vs 2 or 3 vs > 3), and previous bortezomib treatment (no vs yes). The study protocol was approved by an independent ethics committee or institutional review board at each study center and was conducted in accordance with the principles of the Declaration of Helsinki and the International Conference on Harmonisation Good Clinical Practice guidelines. All patients provided written informed consent.

Patients were randomly assigned 1:1 to receive D-Vd or Vd. All patients received eight 21-day cycles of Vd. Bortezomib (1.3 mg/m^2^) was administered subcutaneously on days 1, 4, 8, and 11 during cycles 1 through 8. Dexamethasone (20 mg) was given orally or intravenously on days 1, 2, 4, 5, 8, 9, 11, and 12 during cycles 1 through 8. Daratumumab (16 mg/kg) was administered intravenously to patients in the D-Vd group once weekly during cycles 1 through 3, once every 3 weeks during cycles 4 through 8, and once every 4 weeks thereafter until disease progression. Patients in the Vd group were to receive a maximum of 8 cycles of Vd followed by observation until disease progression; following the primary analysis, patients whose disease progressed could choose to receive daratumumab monotherapy.

### Cytogenetic risk

Cytogenetic risk was evaluated using local FISH or karyotyping. Determination of each abnormality and threshold of frequencies to consider a positive finding was determined locally and varied by site. Patients in the intent-to-treat (ITT) population who had at least 1 FISH or karyotyping assessment were included in the analysis. High-risk patients were defined as having 1 or more of the following cytogenetic abnormalities identified: t(4;14), t(14;16), or del17p.

### MRD evaluation

MRD was evaluated at the time of the suspected CR (including stringent CR; blinded to treatment group) and at 6 and 12 months after the first treatment dose (i.e., at the end of Vd therapy and 6 months later, respectively). Additional MRD evaluations were required every 12 months after CR. MRD was evaluated by next-generation sequencing using the clonoSEQ® assay V2.0 (Adaptive Biotechnologies, Seattle, WA, USA) at a sensitivity threshold of 10^−5^ (1 cancer cell per 100,000 nucleated cells). Patients were considered MRD positive if they had an MRD-positive or indeterminate test result or were not assessed. Sustained MRD negativity was defined as maintenance of MRD negativity at the 10^−5^ sensitivity threshold for at least 6 months or at least 12 months.

### Statistical analyses and assessments

The primary endpoint of the study was PFS. Exploratory analyses were performed for subgroups of patients based on cytogenetic risk status. PFS was assessed in patients in the ITT population who met the biomarker criteria for risk assessment. The response-evaluable analysis set included patients who had measurable disease at the baseline or screening visit and who received at least 1 study treatment and had at least 1 post-baseline disease assessment. The safety population comprised individuals who received at least 1 administration of study treatment.

PFS and time to response were compared between the D-Vd and Vd groups using a stratified log-rank test. A Cox proportional hazards model was used to estimate HRs and 95% CIs, with treatment as the sole explanatory variable. The Kaplan-Meier method was used to estimate distributions. PFS on the subsequent line of therapy (PFS2) was defined as the time from randomization to progressive disease after the next line of subsequent therapy or death. Differences between treatment groups for overall response rates, VGPR or better rates, and CR or better rates were measured by a stratified Cochran-Mantel-Haenszel chi-square test.

Patients in the ITT population who met the biomarker criteria for risk assessment were evaluated for MRD and sustained MRD negativity to allow for stringent and unbiased evaluation. MRD-negativity rates were defined as the proportions of patients achieving MRD-negative status at any time after the first treatment dose and were compared between the D-Vd and Vd treatment groups using a Fisher’s exact test.

## Results

### Patients and treatments

A total of 498 patients were randomized, with 251 assigned to D-Vd and 247 assigned to Vd. A total of 356 (71%) patients underwent cytogenetic testing; 283 (57%) patients were evaluated using FISH, 217 (44%) patients were evaluated using karyotyping, and 144 (29%) were evaluated using both. Of these, 40 (16%) patients in the D-Vd group and 35 (14%) patients in the Vd group had high cytogenetic risk abnormalities. Forty of 158 patients in the D-Vd group and 35 of 173 patients in the Vd group who underwent FISH testing were defined as high risk. Two of 130 patients in the D-Vd group and 1 of 136 patients in the Vd group who underwent karyotype testing were defined as high risk. A total of 141 (56%) patients in the D-Vd group and 140 (57%) patients in the Vd group had standard cytogenetic risk. Patient demographics, baseline disease, and clinical characteristics stratified by cytogenetic risk status are shown in Table 1. Among patients achieving CR or better, MRD was not evaluated in 15 (16%) patients. Overall, 170 (62%) and 50 (68%) patients in the standard and high cytogenetic risk subgroups discontinued the treatment, respectively (Table 2**)**. Among patients who received Vd and discontinued treatment due to progressive disease, 9 of 34 and 2 of 12 patients in the standard and high cytogenetic risk subgroups, respectively, received daratumumab monotherapy as subsequent therapy.

**Table 1:** Patient demographics, baseline disease, and clinical characteristics

	Standard cytogenetic risk^a^		High cytogenetic risk^a,b^	
Characteristic	D-Vd (*n* = 141)	Vd (*n* = 140)	D-Vd (*n* = 40)	Vd (*n* = 35)
Age, years				
Median (range)	64 (40–88)	64 (33–85)	63 (37–79)	59 (37–81)
≥ 75 years, *n* (%)	9 (6)	20 (14)	4 (10)	5 (14)
Sex, *n* (%)				
Male	79 (56)	89 (64)	22 (55)	18 (51)
Race, *n* (%)				
White	123 (87)	123 (88)	33 (83)	31 (89)
Black or African American	9 (6)	2 (1)	1 (3)	1 (3)
Asian	8 (6)	8 (6)	4 (10)	2 (6)
Native Hawaiian or other Pacific Islander	1 (1)	0	0	0
Other	0	1 (1)	0	0
Unknown/not reported	0	6 (4)	2 (5)	1 (3)
ISS stage, *n* (%)^c^				
I	48 (34)	55 (39)	22 (55)	14 (40)
II	57 (40)	56 (40)	11 (28)	16 (46)
III	36 (26)	29 (21)	7 (18)	5 (14)
ECOG performance status score, *n* (%)				
0	54 (38)	64 (46)	16 (40)	15 (43)
1	78 (55)	62 (44)	22 (55)	19 (54)
2	9 (6)	14 (10)	2 (5)	1 (3)
Cytogenetic profile, *n* (%)^a,b^				
t(4;14)	–	–	13 (33)	15 (43)
t(14;16)	–	–	4 (10)	5 (14)
del17p	–	–	27 (68)	20 (57)
≥ 2 risk factors^d^	–	–	4 (10)	4 (11)
Median (range) time from diagnosis, years	4.3 (0.7–20.7)	3.6 (0.6–18.6)	3.3 (1.0–10.5)	3.7 (1.0–14.8)
Prior lines of therapy, *n* (%)				
1	70 (50)	67 (48)	21 (53)	12 (34)
2	32 (23)	40 (29)	11 (28)	15 (43)
3	25 (18)	16 (11)	5 (13)	6 (17)
> 3	14 (10)	17 (12)	3 (8)	2 (6)
Median (range)	2 (1–9)	2 (1–10)	1 (1–6)	2 (1–4)
Prior ASCT, *n* (%)	90 (64)	79 (56)	27 (68)	21 (60)
Prior PI, *n* (%)	101 (72)	94 (67)	27 (68)	28 (80)
Bortezomib	98 (70)	89 (64)	25 (63)	26 (74)
Prior IMiD, *n* (%)	104 (74)	110 (79)	28 (70)	29 (83)
Lenalidomide	52 (37)	66 (47)	15 (38)	17 (49)
Prior PI + IMiD, *n* (%)	73 (52)	67 (48)	15 (38)	22 (63)
Refractory to PI only, *n* (%)	1 (1)	2 (1)	2 (5)	1 (3)
Refractory to IMiD only, *n* (%)	41 (29)	51 (36)	12 (30)	11 (31)
Refractory to PI and IMiD, *n* (%)	6 (4)	2 (1)	0	4 (11)
Refractory to last line of therapy, *n* (%)	40 (28)	48 (34)	11 (28)	14 (40)

*D-Vd* daratumumab/bortezomib/dexamethasone, *Vd* bortezomib/dexamethasone, *ISS* International Staging System, *ECOG* Eastern Cooperative Oncology Group, *ASCT* autologous stem cell transplantation, *PI* proteasome inhibitor, *IMiD* immunomodulatory drug, *FISH* fluorescence in situ hybridization

Note: percentages may not equal 100% due to rounding

^a^Based on FISH/karyotype testing

^b^Patients with high cytogenetic risk had a t(4;14), t(14;16), or del17p abnormality

^c^ISS stage is derived based on the combination of serum β2-microglobulin and albumin

^d^Patients with ≥ 2 of the t(4;14), t(14;16), or del17p risk factors

**Table 2:** Patient disposition based on cytogenetic risk status

	Standard risk^a^		High risk^a,b^	
Treatment discontinuation, *n* (%)^c^	D-Vd (*n* = 137)	Vd (*n* = 136)	D-Vd (*n* = 40)	Vd (*n* = 34)
Patients who discontinued treatment	108 (79)	62 (46)	33 (83)	17 (50)
Reason for discontinuation				
Progressive disease	86 (63)	34 (25)	27 (68)	12 (35)
Adverse event	11 (8)	15 (11)	5 (13)	3 (9)
Noncompliance with study drug^d^	5 (4)	5 (4)	0	1 (3)
Withdrawal by patient	1 (1)	6 (4)	0	1 (3)
Death	2 (1)	2 (1)	1 (3)	0
Physician decision	3 (2)	0	0	0

*D-Vd* daratumumab/bortezomib/dexamethasone, *Vd* bortezomib/dexamethasone, *FISH* fluorescence in situ hybridization

^a^Based on FISH/karyotyping

^b^Patients with high cytogenetic risk had a t(4;14), t(14;16), or del17p abnormality

^c^Safety population

^d^Based on reason “Patient refused to further study treatment” at “End of treatment”

### Updated efficacy results

**Fig. 1:**
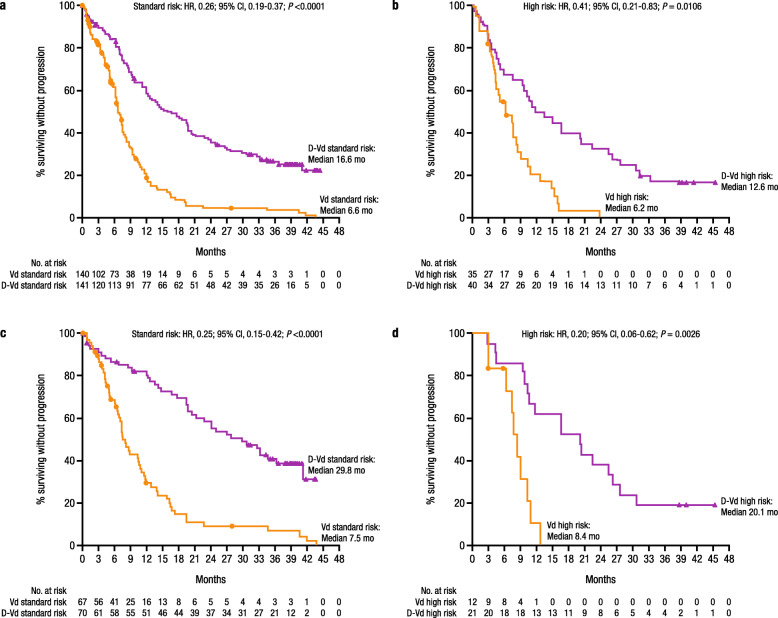
PFS based on cytogenetic risk status. PFS in the ITT/biomarker risk population (patients in the ITT population who met the biomarker criteria for risk assessment): **a** standard cytogenetic risk patients and **b** high cytogenetic risk patients. PFS in patients with 1 prior line of therapy: **c** standard cytogenetic risk patients and **d** high cytogenetic risk patients. CI, confidence interval; D-Vd, daratumumab plus bortezomib/dexamethasone; HR, hazard ratio; ITT intent-to-treat; PFS, progression-free survival; Vd, bortezomib/dexamethasone

After a median follow-up of 40.0 months, treatment with D-Vd prolonged median PFS compared with Vd alone in patients with standard cytogenetic risk (16.6 months vs 6.6 months; HR, 0.26; 95% CI, 0.19–0.37; *P* < 0.0001; Fig. 1a) as well as high cytogenetic risk (12.6 months vs 6.2 months; HR, 0.41; 95% CI, 0.21–0.83; *P* = 0.0106; Fig. 1b) in the ITT population. Among a subset of patients who had received 1 prior line of therapy, treatment with D-Vd prolonged median PFS versus Vd in patients with standard cytogenetic risk (29.8 months vs 7.5 months; HR, 0.25; 95% CI, 0.15–0.42; *P* < 0.0001; Fig. 1c) and high cytogenetic risk (20.1 months vs 8.4 months; HR, 0.20; 95% CI, 0.06–0.62; *P* = 0.0026; Fig. 1d).

Higher overall response rate was achieved with D-Vd versus Vd (standard risk, 84% vs 62%; *P* < 0.0001; high risk, 85% vs 56%; *P* = 0.0512), including deep responses of CR or better (standard risk, 28% vs 10%; high risk, 28% vs 6%) and VGPR or better (standard risk, 62% vs 28%; *P* < 0.0001; high risk, 59% vs 32%; *P* = 0.1259; Table 3). Median time to VGPR or better was decreased with D-Vd compared with Vd in patients with standard cytogenetic risk (3.5 months vs not estimable; HR, 2.16; 95% CI, 1.46–3.20; *P* < 0.0001) and high cytogenetic risk (3.5 months vs 6.2 months; HR, 1.96; 95% CI, 0.86–4.45; *P* = 0.1004).

**Table 3:** Response and MRD-negativity rates in patients with standard and high cytogenetic risk

	Standard risk			High risk^a^		
Response, *n* (%)^b^	D-Vd (*n* = 135)	Vd (*n* = 134)	*P* value	D-Vd (*n* = 39)	Vd (*n* = 34)	*P* value
ORR	113 (84)	83 (62)	< 0.0001	33 (85)	19 (56)	0.0512
≥ CR^c^	38 (28)	13 (10)		11 (28)	2 (6)	
sCR	12 (9)	3 (2)		4 (10)	0	
CR	26 (19)	10 (8)		7 (18)	2 (6)	
≥ VGPR^d^	83 (62)	38 (28)	< 0.0001	23 (59)	11 (32)	0.1259
VGPR	45 (33)	25 (19)		12 (31)	9 (27)	
PR	30 (22)	45 (34)		10 (26)	8 (24)	
MRD negative (10^−5^)^e^						
*n* (%)	16 (11)	4 (3)	0.0091	6 (15)	0	0.0271
Sustained MRD negativity (≥ 6 months), *n* (%)	9 (6)	3 (2)	0.1374	5 (13)	0	0.0569
Sustained MRD negativity (≥ 12 months), *n* (%)	2 (1)	0	0.4982	3 (8)	0	0.2432

*CR* complete response, *D-Vd* daratumumab plus bortezomib/dexamethasone, *ITT* intent-to-treat*, MRD* minimal residual disease, *ORR* overall response rate, *PR* partial response, *sCR* stringent complete response, *Vd* bortezomib/dexamethasone, *VGPR* very good partial response

^a^Patients with high cytogenetic risk had a t(4;14), t(14;16), or del17p abnormality

^b^Response-evaluable population

^c^≥ CR = sCR + CR

^d^≥ VGPR = sCR + CR + VGPR

^e^ITT population (standard risk: D-Vd, *n* = 141; Vd, *n* = 140; high risk: D-Vd, *n* = 40; Vd, *n* = 35)

Rates of MRD negativity at the 10^−5^ sensitivity threshold were higher with D-Vd compared with Vd in patients with standard cytogenetic risk (11% vs 3%; *P =* 0.0091) and high cytogenetic risk (15% vs 0%; *P* = 0.0271; Table 3). MRD negativity was sustained for at least 6 months in a greater number of patients treated with D-Vd versus Vd, regardless of cytogenetic risk status. MRD negativity was sustained for at least 12 months in 2 (1%) patients with standard cytogenetic risk and 3 (8%) patients with high cytogenetic risk in the D-Vd group compared with none in both cytogenetic risk categories in the Vd group.

**Fig. 2:**
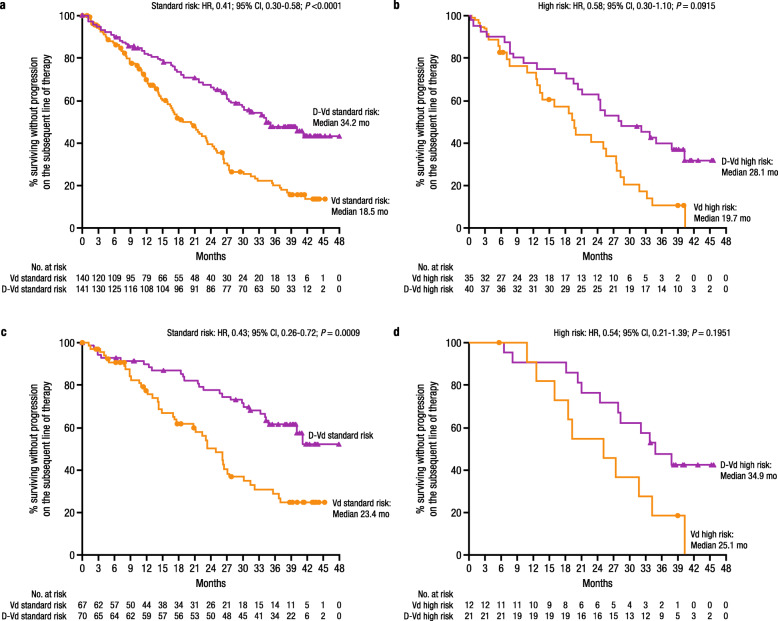
PFS2 based on cytogenetic risk status. PFS2 in the ITT/biomarker risk population (patients in the ITT population who met the biomarker criteria for risk assessment): **a** standard cytogenetic risk patients and **b** high cytogenetic risk patients. PFS2 in patients with 1 prior line of therapy: **c** standard cytogenetic risk patients and **d** high cytogenetic risk patients. CI, confidence interval; D-Vd, daratumumab plus bortezomib/dexamethasone; HR, hazard ratio; ITT intent-to-treat; PFS2, progression-free survival on the subsequent line of therapy; Vd, bortezomib/dexamethasone

Median PFS2 was prolonged with D-Vd compared with Vd in the standard cytogenetic risk (34.2 months vs 18.5 months; HR, 0.41; 95% CI, 0.30–0.58; *P* < 0.0001; Fig. 2a) and high cytogenetic risk (28.1 months vs 19.7 months; HR, 0.58; 95% CI, 0.30–1.10; *P* = 0.0915; Fig. 2b) subgroups. Among the subset of patients with 1 prior line of therapy, median PFS2 was prolonged with D-Vd versus Vd in patients with standard cytogenetic risk (not estimable vs 23.4 months; HR, 0.43; 95% CI, 0.26–0.72; *P* = 0.0009; Fig. 2c). For patients with high cytogenetic risk, median PFS2 was prolonged with D-Vd versus Vd (34.9 months vs 25.1 months; HR, 0.54; 95% CI, 0.21–1.39; *P* = 0.1951; Fig 2d).

At the time of the analysis, among patients with high cytogenetic risk, 21 deaths were observed in the D-Vd group versus 23 deaths in the Vd group. The overall survival data are immature and follow-up for overall survival is ongoing.

### Updated safety results

The most frequent any grade treatment-emergent adverse events observed in at least 25% of patients and the most frequent grade 3/4 treatment-emergent adverse events observed in at least 5% of patients are summarized in Table 4. Treatment discontinuation rates due to treatment-emergent adverse events were similar between treatment groups for patients with standard cytogenetic risk (11 [8%] patients in the D-Vd group and 14 [10%] of patients in the Vd group) and among patients with high cytogenetic risk (4 [10%] patients in the D-Vd group and 3 [9%] patients in the Vd group).

**Table 4:** Most common any-grade (≥ 25% of patients) and grade 3/4 (≥ 5% of patients) TEAEs

	Any grade				Grade 3/4			
	Standard risk		High risk^a^		Standard risk		High risk^a^	
TEAE, *n* (%)	D-Vd (*n* = 137)	Vd (*n* = 136)	D-Vd (*n* = 40)	Vd (*n* = 34)	D-Vd (*n* = 137)	Vd (*n* = 136)	D-Vd (*n* = 40)	Vd (*n* = 34)
Hematologic								
Thrombocytopenia	85 (62)	58 (43)	24 (60)	16 (47)	65 (47)	45 (33)	19 (48)	12 (35)
Anemia	45 (33)	41 (30)	7 (18)	14 (41)	25 (18)	23 (17)	4 (10)	6 (18)
Neutropenia	29 (21)	16 (12)	9 (23)	3 (9)	21 (15)	6 (4)	6 (15)	2 (6)
Lymphopenia	18 (13)	5 (4)	4 (10)	4 (12)	14 (10)	3 (2)	3 (8)	3 (9)
Leukopenia	15 (11)	5 (4)	3 (8)	3 (9)	5 (4)	1 (1)	1 (3)	2 (6)
Nonhematologic								
Peripheral sensory neuropathy	67 (49)	50 (37)	22 (55)	13 (38)	4 (3)	8 (6)	2 (5)	4 (12)
Upper respiratory tract infection	43 (31)	20 (15)	15 (38)	8 (24)	1 (1)	0	3 (8)	1 (3)
Diarrhea	42 (31)	35 (26)	11 (28)	6 (18)	6 (4)	2 (2)	1 (3)	0
Cough	40 (29)	19 (14)	9 (23)	4 (12)	0	0	0	0
Fatigue	25 (18)	40 (29)	17 (43)	8 (24)	6 (4)	5 (4)	2 (5)	1 (3)
Back pain	24 (18)	15 (11)	13 (33)	1 (3)	3 (2)	0	1 (3)	0
Insomnia	22 (16)	20 (15)	11 (28)	5 (15)	2 (2)	0	0	1 (3)
Pneumonia	22 (16)	20 (15)	5 (13)	4 (12)	15 (11)	14 (10)	2 (5)	3 (9)
Asthenia	15 (11)	19 (14)	4 (10)	9 (27)	1 (1)	3 (2)	0	1 (3)
Hypertension	15 (11)	5 (4)	4 (10)	1 (3)	9 (7)	1 (1)	2 (5)	0
Decreased appetite	14 (10)	8 (6)	10 (25)	1 (3)	0	1 (1)	1 (3)	0
Spinal pain	4 (3)	3 (2)	2 (5)	0	1 (1)	0	2 (5)	0
Gastroenteritis	2 (2)	3 (2)	2 (5)	1 (3)	0	2 (2)	2 (5)	1 (3)
Squamous cell carcinoma of the skin	0	0	2 (5)	0	0	0	2 (5)	0

*TEAE* treatment-emergent adverse event, *D-Vd* daratumumab/bortezomib/dexamethasone, *Vd* bortezomib/dexamethasone

^a^Patients with high cytogenetic risk had a t(4;14), t(14;16), or del17p abnormality

## Discussion

After a median follow-up of more than 3 years, D-Vd continued to demonstrate substantially improved efficacy in terms of PFS compared with Vd alone in patients with RRMM, regardless of cytogenetic risk status. D-Vd reduced the risk of disease progression or death by 74% versus Vd alone in patients with standard cytogenetic risk and by 59% in patients with high cytogenetic risk. Among patients treated with D-Vd, median PFS was 16.6 months in patients with standard cytogenetic risk (vs 6.6 months with Vd; *P* < 0.0001) and 12.6 months in patients with high cytogenetic risk (vs 6.2 months with Vd; *P* = 0.0106). The PFS benefit of D-Vd over Vd was especially pronounced in the subset of patients who received 1 prior line of therapy, reducing the risk of disease progression or death by 75% and 80% in patients with standard and high cytogenetic risk, respectively. D-Vd achieved deep responses compared with Vd, with higher rates of VGPR or better and CR or better, regardless of cytogenetic risk status. Rates of MRD negativity (10^−5^ sensitivity threshold) were higher with D-Vd versus Vd in patients with standard cytogenetic risk (11% vs 3%; *P* = 0.0091) and high cytogenetic risk (15% vs 0%; *P* = 0.0271). Moreover, sustained MRD-negative responses were observed in more patients treated with D-Vd compared with Vd regardless of cytogenetic risk status. D-Vd prolonged median PFS2 versus Vd alone in both cytogenetic risk subgroups. Overall, improved outcomes were achieved by D-Vd versus Vd in patients with high cytogenetic risk, but clinical benefits were of lesser magnitude than D-Vd in patients with standard cytogenetic risk.

The safety profile of D-Vd in standard and high cytogenetic risk subgroups was consistent with the overall population of CASTOR. No new safety signals were identified.

The results reported here after extended follow-up further strengthen results reported after a median follow-up of 13.0 months [18]. In this earlier analysis in patients with high cytogenetic risk, PFS was prolonged with D-Vd versus Vd (median 11.2 months vs 7.2 months; HR, 0.49; 95% CI, 0.27–0.89; *P* = 0.0167). With a median follow-up of 40.0 months, the efficacy of D-Vd versus Vd in these high-risk patients was maintained, with prolonged median PFS (HR, 0.41) and higher MRD-negativity rates (15% vs 0%; *P* = 0.0271) in this difficult-to-treat patient population.

While cross-trial comparisons should be approached with caution, especially due to lack of consensus on thresholds for risk groups, the efficacy of D-Vd in patients with high cytogenetic risk appears favorable to that reported in other studies of proteasome inhibitor–containing regimens in RRMM (Table 5). In a pre-planned subgroup analysis of the ENDEAVOR study based on baseline cytogenetic risk, carfilzomib in combination with dexamethasone reduced the risk of disease progression or death by 35% versus Vd in patients with high-risk RRMM (defined as t[4;14] or t[14;16] in ≥ 10% of screened plasma cells or del17p in ≥ 20% of screened plasma cells assessed by FISH) [19]. Median PFS was 8.8 months with carfilzomib in combination with dexamethasone versus 6.0 months with Vd in patients with high cytogenetic risk (HR, 0.65; 95% CI, 0.45–0.92; *P* = 0.0075) and CR or better rates were 16% versus 4%, respectively. In a pre-planned subgroup analysis of the phase 3 ASPIRE study, among patients with high cytogenetic risk (t[4;14], t[14;16], or del17p in ≥ 60% of screened plasma cells assessed by FISH), median PFS was 23.1 months with carfilzomib plus Rd versus 13.9 months with Rd alone (HR, 0.70; 95% CI, 0.43–1.16; *P* = 0.0829) [20]. In the phase 3 PANORAMA-1 trial of panobinostat plus Vd versus Vd alone in RRMM, the HR for median PFS in high-risk patients (t[4;14], t[14;16], or del17p assessed by FISH) was 0.47 (95% CI, 0.18–1.25) in favor of panobinostat-Vd [21].

**Table 5:** Summary of median PFS of high cytogenetic risk patients with RRMM in randomized, phase 3 trials

Trial name	High cytogenetic risk definition	Arm 1 (*n*)	Arm 2 (*n*)	Arm 1 median PFS, months	Arm 2 median PFS, months	Hazard ratio (95% CI); *P* value
CASTOR	t(4;14), t(14;16), or del17p assessed by FISH or karyotyping	D-Vd (40)	Vd (35)	12.6	6.2	0.41 (0.21–0.83); 0.0106
ENDEAVOR [19]	t(4;14) or t(14;16) in ≥ 10% of screened plasma cells or del17p in ≥ 20% of screened plasma cells assessed by FISH	Kd (97)	Vd (113)	8.8	6.0	0.65 (0.45–0.92); 0.0075
ASPIRE [20]	t(4;14), t(14;16), or del17p (in ≥ 60% of screened plasma cells) assessed by FISH	KRd (48)	Rd (52)	23.1	13.9	0.70 (0.43–1.16); 0.0829
PANORAMA-1 [21]	t(4;14), t(14;16), or del17p assessed by FISH	Panobinostat plus Vd	Vd	–	–	0.47 (0.18–1.25)

*CI* confidence interval, *D-Vd* daratumumab plus bortezomib/dexamethasone, *FISH* fluorescence in situ hybridization*, Kd* carfilzomib/dexamethasone*, KRd* carfilzomib/lenalidomide/dexamethasone, *PFS* progression-free survival, *Rd* lenalidomide/dexamethasone, *Vd* bortezomib/dexamethasone

The efficacy of daratumumab plus standard of care, regardless of cytogenetic risk status, was also demonstrated in the phase 3 POLLUX study of daratumumab plus Rd versus Rd alone in RRMM [22]. At a median follow-up of more than 3 years, median PFS was prolonged with daratumumab plus Rd (D-Rd) versus Rd in patients with standard (not reached vs 19.9 months; HR, 0.41; 95% CI, 0.31–0.55; *P* < 0.0001) and high (26.8 vs 8.8 months; HR, 0.54; 95% CI, 0.32–0.91; *P* = 0.0175) cytogenetic risk, and deep responses were achieved with D-Rd in both cytogenetic risk subgroups. It is noteworthy that D-Vd and D-Rd, but not Vd nor Rd, achieved MRD negativity in patients with high cytogenetic risk, which suggests that targeting CD38 in combination with other standard of care regimens helps improve the historically poor outcomes observed in this patient population [23–27]. Looking ahead, there continues to be a gap in treatment options for high-risk patients with RRMM; potential treatment regimens that can be studied include daratumumab in combination with pomalidomide, carfilzomib, or bortezomib, lenalidomide, and dexamethasone.

This report is limited by incomplete cytogenetic abnormality data collected for patients enrolled in the CASTOR study; cytogenetic testing was not performed in 29% of patients in the study. Cytogenetic testing was performed locally and no per-protocol specific cut-off values were used for defining the presence of genetic abnormalities; cut-off values used at each site were not collected. Additionally, MRD was not assessed in patients with VGPR and in 16% of patients with CR or better. Of patients with available cytogenetic abnormality data, patients without MRD assessment were considered MRD positive, potentially underestimating the rate of MRD negativity. Lastly, small sample sizes in the cytogenetic risk subgroups precluded us from conducting a multivariate analysis to account for baseline differences.

## Conclusions

In this subgroup analysis, D-Vd demonstrated a clear efficacy benefit compared with Vd in patients with RRMM and high cytogenetic risk in CASTOR. When combined with the recently updated POLLUX results, these findings reinforce the effectiveness and tolerability of daratumumab plus standard of care as a treatment for MM, regardless of cytogenetic risk status.

## Data Availability

The data sharing policy of Janssen Pharmaceutical Companies of Johnson & Johnson is available at https://www.janssen.com/clinical-trials/transparency. As noted on this site, requests for access to the study data can be submitted through Yale Open Data Access (YODA) Project site at http://yoda.yale.edu.

## Abbreviations

CI: Confidence interval
CR: Complete response
D-Rd: Daratumumab plus lenalidomide/dexamethasone
D-Vd: Daratumumab plus bortezomib/dexamethasone
FISH: Fluorescence in situ hybridization
HR: Hazard ratio
ITT: Intent-to-treat
MRD: Minimal residual disease
MM: Multiple myeloma
PFS: Progression-free survival
PFS2: Progression-free survival on the subsequent line of therapy
Rd: Lenalidomide/dexamethasone
RRMM: Relapsed or refractory multiple myeloma
Vd: Bortezomib/dexamethasone
VGPR: Very good partial response
